# Folic Acid Supplementation and Risk of Gestational Diabetes Mellitus: A Systematic Review of the Literature

**DOI:** 10.3390/ijms26167977

**Published:** 2025-08-18

**Authors:** Alejandro Salvador Gómez-Cabrera, Ana Elizabeth González-Santiago, Rolando Castañeda-Arellano, Fernanda Isadora Corona-Meraz, Raúl Cuauhtemoc Baptista-Rosas, María Guadalupe Sánchez-Parada

**Affiliations:** 1 Departamento de Ciencias Biomédicas, División de Ciencias de la Salud, Centro Universitario de Tonalá, Universidad de Guadalajara, Tonalá 45425, Jalisco, Mexico; alejandro.gomez@academicos.udg.mx (A.S.G.-C.); ana.gonzalez@academicos.udg.mx (A.E.G.-S.); rolando.castaneda@academicos.udg.mx (R.C.-A.); mariaf.corona@academicos.udg.mx (F.I.C.-M.); 2 Departamento de Ciencias de la Salud-Enfermedad como Proceso Individual, División de Ciencias de la Salud, Centro Universitario de Tonalá, Universidad de Guadalajara, Tonalá 45425, Jalisco, Mexico; raul.baptista@academicos.udg.mx; 3 Unidad de Intervención de Medicina Crítica, Hospital General de Occidente, Secretaría de Salud Jalisco, Zapopan 45170, Jalisco, Mexico

**Keywords:** gestational diabetes mellitus, insulin resistance, folic acid supplementation, homocysteine, vitamin B12

## Abstract

Gestational diabetes mellitus (GDM) affects approximately 14% of pregnancies globally and has been hypothesized to be influenced by periconceptional and early pregnancy folic acid (FA) supplementation, a practice recommended to prevent neural tube defects. To evaluate this association, we conducted a systematic review of studies published between 2015 and 2024 examining FA use and GDM risk. Twelve studies met the inclusion criteria, including ten cohort studies and two case-control studies. While findings were mixed, several prospective studies suggested that high daily FA intake (≥800 μg) or prolonged use (>3–6 months) may be associated with increased odds of GDM, especially when initiated preconceptionally. Conversely, standard-dose supplementation (≈400 μg) appeared neutral or potentially protective in some populations. Notably, high folate status combined with low vitamin B12 was linked to increased GDM risk, suggesting metabolic interaction. Overall, most studies were of moderate to high methodological quality. Although current evidence is inconclusive, these results support cautious use of high-dose FA supplementation and the importance of individualized prenatal nutrition, particularly considering B12 status. Further research is needed to clarify biological mechanisms.

## 1. Introduction

**Figure 1:**
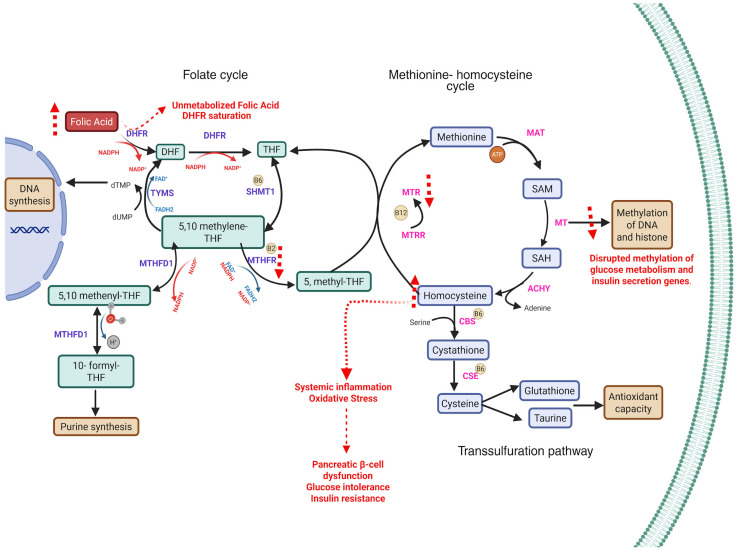
Disruptions in one-carbon metabolism due to folate and vitamin B12 imbalance and their implications for gestational diabetes mellitus (GDM). The diagram outlines the folate cycle, methionine–homocysteine cycle, and transsulfuration pathway, highlighting the key enzymes and metabolites. Excess folic acid (FA) intake can lead to accumulation of unmetabolized folic acid (UMFA) and dihydrofolate (DHF), resulting in DHFR (dihydrofolate reductase) saturation and impaired formation of tetrahydrofolate (THF). This limits the availability of 5-methyltetrahydrofolate (5-methyl-THF), a methyl donor required by methionine synthase (MTR) for the remethylation of homocysteine (Hcy) to methionine in a vitamin B12-dependent reaction [22]. Elevated homocysteine may promote oxidative stress, systemic inflammation, pancreatic β-cell dysfunction, glucose intolerance, and insulin resistance [23,24]. While not depicted, hormonal changes during pregnancy (progesterone, leptin, adiponectin) may also contribute to insulin resistance. Red dashed arrows indicate disruptions specifically attributed to excessive folic acid exposure. Abbreviations: FA, folic acid; DHF, dihydrofolate; THF, tetrahydrofolate; SHMT1, serine hydroxymethyltransferase 1; 5,10-methylene-THF, 5,10-methylenetetrahydrofolate; 5,10-methenyl-THF, 5,10-methenyltetrahydrofolate; 10-formyl-THF, 10-formyltetrahydrofolate; MTHFD1, methylenetetrahydrofolate dehydrogenase 1; MTHFR, methylenetetrahydrofolate reductase; 5-methyl-THF, 5-methyltetrahydrofolate; MTRR, methionine synthase reductase; SAM, S-adenosylmethionine; SAH, S-adenosylhomocysteine; CBS, cystathionine β-synthase; CSE, cystathionine γ-lyase; MAT, methionine adenosyltransferase; MT, methyltransferase; TYMS, thymidylate synthase; NADPH, nicotinamide adenine dinucleotide phosphate (reduced form).

Gestational diabetes mellitus (GDM), defined as glucose intolerance first recognized during pregnancy [1], affects approximately 14% of pregnancies worldwide [2]. Its rising prevalence parallels global increases in obesity and type 2 diabetes, posing significant risks for both mother and child. The most common complications include an increased likelihood of requiring a cesarean section [3], preeclampsia [4], and a higher risk of progressing to type 2 diabetes mellitus (T2DM), although GDM resolves in most cases after delivery [5]. For the offspring it has been reported an increased likelihood of macrosomia [6] hypoglycemia [3,7], a higher incidence of congenital malformations [8], respiratory distress [9], lower Apgar scores at one and five minutes, and low birth weight [10] as well as higher T2DM risk later in life [11]. Identifying modifiable risk factors for GDM remains a key public health objective. One area of emerging interest is the potential metabolic impact of folic acid (FA) supplementation, a universally recommended intervention to prevent neural tube defects. Current guidelines advise 400 μg/day of FA from preconception through early pregnancy, with higher doses (5 mg/day) for high-risk women [12,13,14]. Beyond its neuroprotective role, folate participates in one-carbon metabolism and DNA methylation, suggesting broader physiological effects as explained by Figure 1 [15]. Recent studies have raised concerns that excessive FA intake may influence glucose metabolism, particularly when vitamin B12 deficiency coexists, potentially leading to insulin resistance through mechanisms like the methylfolate trap or immune-mediated pathways [16,17,18]. However, epidemiological findings are conflicting: while some studies associate high-dose or prolonged FA use with increased GDM risk, others report no association or even a protective effect [19,20,21]. To address this controversy, we systematically reviewed recent human studies on FA supplementation and GDM. Our goal was to clarify the strength and direction of the association, explore potential biological mechanisms, and consider implications for prenatal nutritional recommendations.

## 2. Methods

This search protocol was designed following the Preferred Reporting Items for Systematic Reviews and Meta-Analyses (PRISMA) [25] guidelines to ensure a comprehensive and transparent search of relevant literature.

### 2.1. Study Design

To identify relevant studies on the effect of folic acid consumption in GDM events, a systematic search was conducted in various electronic databases.

### 2.2. Eligibility Criteria

The search strategy was designed to capture relevant studies investigating the relationship between gestational diabetes mellitus and folic acid, as well as the effects of folic acid supplementation. The search was limited to studies published in the last 10 years and restricted to articles in English due to resource and time limitations. Randomized controlled trials, observational studies (cohorts, case-control) were included in the review. The primary outcomes were exposure to FA and GDM risk. We excluded studies that did not directly address the relationship between folic acid and GDM, as well as non-human studies, case reports, editorials, letters to the editor, and articles for which the full text was not available. To ensure high methodological quality and scientific rigor, the research team also agreed to exclude studies published in journals not listed in the Journal Citation Report (JCR). Although Web of Science provides access to JCR-indexed journals, we included PubMed and Scopus in the initial search strategy to enhance sensitivity and ensure broader coverage. This approach allowed us to capture potentially relevant studies that could later be screened for inclusion based on JCR indexing status.

### 2.3. Literature Search and Selection of Articles

The databases PubMed, Scopus, and Web of Science were used for this search. The search terms used included a combination of keywords and Medical Subject Headings (MeSH) related to gestational diabetes mellitus, folic acid, and supplementation. Boolean operators “AND” and “OR” were used to combine search terms appropriately. The search strategy was implemented using a combination of search terms, including variations of “gestational diabetes mellitus” AND “folic acid supplementation” AND “risk”, “gestational diabetes” AND “folic acid” AND “supplementation”, “GDM” AND “folic acid intake” AND “risk factor”, “diabetes in pregnancy” AND “folate supplementation” AND “association”, “pregnancy-induced diabetes” AND “folic acid” AND “increased risk”, “pregnancy-related diabetes” AND “folic acid use” AND “GDM incidence”, “gestational diabetes mellitus” AND “folate intake” AND “odds of GDM”, “hyperglycemia in pregnancy” AND “folic acid status” AND “glucose intolerance”, “gestational diabetes” AND “unmetabolized folic acid” AND “insulin resistance”, “GDM” AND “high-dose folic acid” AND “development of GDM”. Additionally, the reference lists of relevant articles found during the initial search were manually reviewed to identify additional studies that may have been missed.

**Figure 2:**
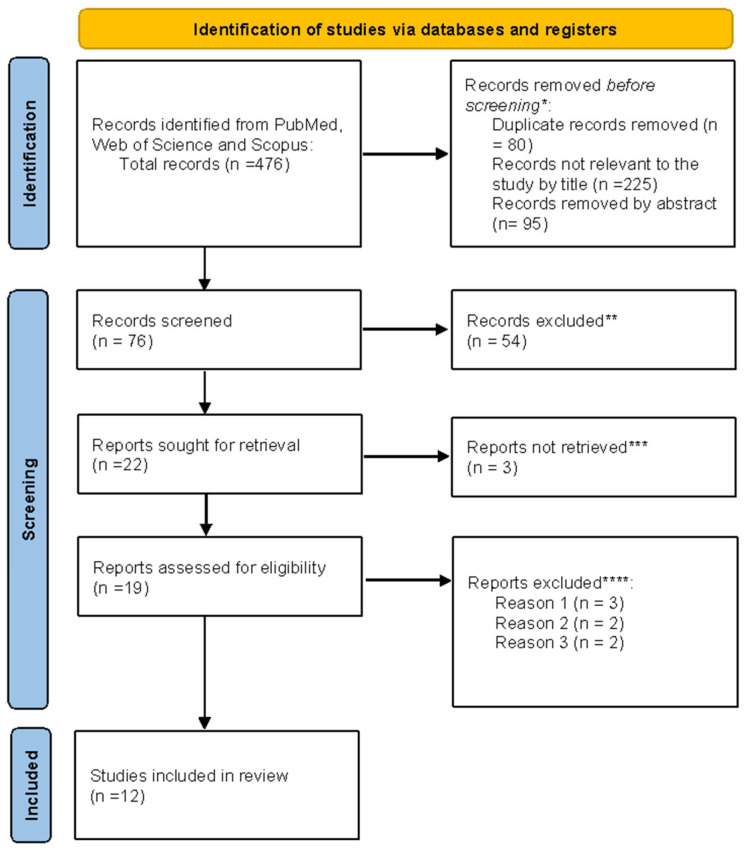
PRISMA flowchart. * Studies not relevant to the present review ** No automation tools were used. All studies were assessed individually by a researcher. *** Unable to find full text. **** Reason 1: Study on animals. Reason 2: Wrong setting. Reason 3: The research question was not relevant.

All search results were recorded, and duplicates were removed. Identified studies underwent a selection process based on predefined inclusion and exclusion criteria to ensure the relevance and quality of studies included in the systematic review. Figure 2 shows the selection process.

### 2.4. Data Extraction

Twelve articles met the inclusion criteria and were chosen for further data extraction. Data extracted from each article included: title, study design, population characteristics (ethnicity, sample size), intervention details (folic acid dosage, duration, period), effect of FA, risk ratio, and odds ratio reported by the study, as well as key findings.

### 2.5. Risk of Bias Assessment Across Studies

Three authors independently evaluated the studies, and any differences among the authors were discussed by the investigation group until consensus was reached. The methodological quality of the included studies was evaluated using the Newcastle–Ottawa Scale (NOS) [26], which assesses three domains: selection (four items), comparability (two items), and outcome (three items). Based on NOS criteria, studies receiving scores between 7 and 10 were categorized as high quality; low risk of bias, those scoring between 3 and 6 as moderate quality; moderate risk of bias, and those with lower scores as low quality; high risk of bias. In the present review, studies that obtained 6 or more stars were considered to be of high methodological quality and were reported as low risk of bias accordingly.

### 2.6. Synthesis of Results

Given substantial heterogeneity in study designs and reported measures, a meta-analysis was not performed. Instead, we carried out a narrative synthesis. We grouped the findings based on the nature of folic acid exposure (supplementation duration, dose, or timing relative to pregnancy) and whether the study suggested increased risk, decreased risk, or no effect on GDM. We also considered population differences that might explain discrepancies.

## 3. Results

As shown in Figure 2, a total of 476 records were identified through database searching (PubMed, Web of Science, and Scopus). After the removal of 80 duplicate records and exclusion of 225 titles and 95 abstracts that were not relevant to the research question, 76 records remained for screening. Following the title and abstract review, 54 articles were excluded. Of the 22 reports selected for full-text retrieval, 3 could not be obtained. Nineteen full-text articles were assessed for eligibility, of which five were excluded for predefined reasons (*n* = 3: animal studies; *n* = 2: inappropriate setting, 2 = research question not relevant). Ultimately, 12 studies met the inclusion criteria and were incorporated into the final synthesis. All stages of screening and selection were performed manually without automation tools.

### 3.1. Study Characteristics

As shown in Table 1, we included 12 studies, 9 were prospective cohort studies [20,27,28,29,30,31,32,33,34], 2 were case-control studies [35,36], and 1 was a retrospective cohort study [19]. Regarding population origin, 11 studies were conducted in Chinese populations, while 1 study involved Nordic populations from Norway and Sweden [19]. 9 out of 12 studies used the 75-g OGTT as suggested by the International Association of Diabetes and Pregnancy Study Groups [37]. The included studies displayed a wide variation in sample size, ranging from as few as 162 participants [36] to over 1.9 million pregnancies in population-based cohorts [19]. Most studies enrolled between 300 and 24,000 participants, with typical sample sizes around 2000 to 4000 in Chinese prospective cohorts [31,32,33,34]. Regarding methodological quality, six studies were assessed as having low risk of bias [19,28,29,30,35,36]. These studies generally featured rigorous designs and appropriate adjustment for confounders. In contrast, five studies were rated as having moderate risk of bias due to issues such as incomplete exposure characterization or limited statistical adjustment [20,27,29,32,33] none of the studies were judged to have high risk of bias, indicating an overall moderate to high methodological quality among the evidence included.

### 3.2. Folic Acid Supplementation and GDM

The association between FA-supplemented women and their risk for GDM was not consistent in the studies analyzed (Table 1). Some of them showed increased risk when FA was used in high doses or for long periods of time. For example, in the case of Li et al. [31], women who were supplemented with ≥ 800 μg/day for 3 or more months had a higher risk for GDM (aOR = 1.70, 95% CI: 1.30–3.36). Zhu et al. [36] also found that both not using FA (aOR = 7.25, 95% CI: 1.34–39.36) and using > 800 μg/day (aOR = 4.20, 95% CI: 1.03–17.22) were associated with increased risk compared to < 400 μg/day. Huang et al. [29] described something similar, but in a U-shape form, meaning that both no use and use for over 90 days increased the risk (OR = 3.45, 95% CI: 1.01–11.8). Early use in pregnancy during the first trimester was also associated with a higher risk, as reported by Zhu et al. [27] (OR 2.25 95% CI: 1.35–3.76). Lai et al. [28] reported that when folate was high and B12 was low, the risk was also higher (OR = 1.97, 95% CI: 1.05–3.68).

Not all studies showed a positive correlation. In contrast, some studies found protective effects or no relationship. Li et al. [35] reported that when the FA dose was given based on MTHFR/MTRR genotype, GDM incidence dropped to 0.27% in the genotype-tailored dossification group versus 3.24% in the control group; this difference reached statistical significance. Chen et al. [32] found that FA use was linked with a lower GDM risk (OR 0.82, 95% CI: 0.70–0.95). Guo et al. [20] and Zheng et al. [34] reported no association between FA and GDM risk. However, Zheng et al. [34] identified increased GDM risk with higher early pregnancy UMFA ≥P75 (aOR 1.36, 95% CI: 1.01–1.84) and ≥P90 (aOR 1.82, 95%, CI: 1.23–2.69), and Hcy ≥P75 (aOR 1.40, 95% CI: 1.04–1.88). In the Nordic cohort [19], self-reported use had minimal impact (OR 1.10, 95% CI: 1.06–1.14 in Norway, OR 0.89, 95% CI: 0.85–0.93 in Sweden), whereas prescribed 5 mg/day was linked with increased GDM risk (Norway OR 1.33, 95% CI: 1.15–1.53 and Sweden OR 1.56, 95% CI: 1.41–1.74). Cheng et al. [30] reported that FA supplementation ≥3 months before pregnancy increased GDM risk (ARR 1.72, 95% CI: 1.17–2.53); however, FA intake during pregnancy for ≥3 months had no association (ARR 0.92, 95% CI: 0.52–1.65). Li et al. [33] found that the effect varied according to timing and maternal characteristics; in obese women, both sufficient and deficient FA intake were associated with increased GDM risk (aORs 3.57. 95% CI: 2.02–6.34 and 10.82, 95% CI: 1.69–69.45, respectively), stratification by FA intake duration in obese women indicated a potential interaction when taken for less than 3 months, although this did not reach statistical significance (ROR = 1.63, 95% CI: 0.37–7.04).

## 4. Discussion

In this review, findings were mixed between FA supplementation and GDM risk. Evidence suggests that high-dose folic acid (≥800 μg/day) is associated with increased GDM risk, especially with prolonged use preconceptionally (≥3 months) [31,36]. In line with this, the Nordic cohort showed that 5mg/day doses were consistently linked to higher GDM risk [19]. The risk for GDM was further increased when mothers were obese (BMI ≥ 25 kg/m^2^) [27]. U-shaped association was observed with no FA supplementation or high-dose (≥800 μg/day) or long consumption duration (≥3 months) and elevated GDM risk [29,36].

Studies indicate that periconceptional folic acid supplementation is either neutral or mildly protective at the recommended guideline dose (400–800 μg/day) [38]; nonetheless, continuation in mid-pregnancy may increase GDM odds [30,31]. A protective effect was also reported where FA supplementation was individualized by MTHFR and MTRR single-nucleotide variants (SNVs) [35]; however, Zheng et al. found no association with folic acid-related genotypes [34].

A significant finding was that the higher risk for GDM was observed when a high level of folate in serum coexisted with low cobalamin (B12) status [28]. One more study reported a link between high levels of UMFA as well as Hcy in early pregnancy and GDM risk (aOR 1.82, 95%, CI: 1.23–2.69 and aOR 1.40, 95% CI: 1.04–1.88, respectively) [34]. In another study, RBC folate concentrations were measured in GDM and non-GDM pregnancies; notably, high folate concentrations were associated with GDM risk in the second trimester (OR = 2.17, 95% CI = 1.20–3.95) [39], which in turn provides empirical evidence for the anticipated biological mechanisms. Nevertheless, in a 2021 systematic review that included 12 studies about B12 and folate levels in pregnancy reported no association between serum folate and GDM risk, and a “conflicting” association between GDM risk and B12 deficiency [40].

### 4.1. Folic Acid and Potential Biological Mechanisms for GDM

Excessive FA intake has been found to disrupt one-carbon metabolism. It has been reported that high folate levels and B12 deficiency disrupted 1C metabolism; these findings are supported by increased total homocysteine and methylmalonic acid levels [41]. This is consistent with findings in the Folic Acid Clinical Trial, where high-dose folate acid intake increased folate serum level but no other biomarkers for one-carbon metabolism [42], which might in turn confirm some interdependency shown by other studies between FA and B12 in one-carbon metabolism disruption [28,43,44].

Evidence shows a correlation between FA intake and an increased risk for GDM. Numerous studies show that women supplementing with a ≥800 μg/day of FA, especially preconceptionally and in early pregnancy, have elevated odds of developing GDM [24,27,29,31]. Saravanan et al. [18] observed that higher folate concentrations in early pregnancy were associated with an increased risk of GDM, with each 12 nmol/L increase (1 SD) linked to an adjusted relative risk of 1.11 (95% CI: 1.036–1.182). Another study reported that individuals with GDM had significantly higher mean serum folate levels than non-GDM counterparts (37.6 ± 8 vs. 31.9 ± 11.2 nmol/L, *p* = 0.007) [45].

At least 60% of 193 countries mandate folic acid fortification [46]. In pregnant women who come from countries that mandate FA fortification and undergo folic acid supplementation, some studies show levels of unmetabolized folic acid (UMFA) were consistently higher [47,48,49]. The enzyme responsible for catalyzing the conversion from FA to dihydrofolate (DHF) and then to tetrahydrofolate (THF active form) is dihydrofolate reductase (DHFR). It has been reported that the function of DHFR in humans is slow and inadequate, as it becomes saturated when FA intake is higher than 331 μg, leaving UMFA levels increased [50]. Another study showed that even a 200 μg FA dose left measurable UMFA in serum after more than a few hours [51]. Therefore, a higher intake of FA could lead to an accumulation of UMFA as well as higher DHF levels; nevertheless, the UMFA role in GDM remains unclear.

High DHF levels from excessive folate supplementation may downregulate MTHFR, shifting folate metabolism from remethylating Hcy to nucleotide synthesis [52]. This shift might elevate Hcy levels. Elevated Hcy levels are consistently observed in pregnancies affected by GDM [53,54,55] and have been linked to oxidative stress, placental dysfunction [56,57], and impaired insulin secretion [58]. Interestingly, prolonged exposure to Hcy has been linked to reversible diminished pancreatic beta-cell function related to insulinotropic molecules [59]. It has also been proven that Hcy can reduce insulin secretion without beta-cell damage, even though it’s known for its cytotoxic effects [60]. Another potential mechanism by which Hcy levels may be relevant to maternal insulin resistance is through the modification of the immature form of the insulin receptor (pro-IR) via cysteine-homocysteinylation (C-Hcy) at the cysteine-825 residue. This modification has been shown to reduce mature insulin receptor levels across multiple tissues in mice [61]. High oxidative stress from Hcy effects may impair GLUT4 translocation [62], as well as endothelial damage [63]. High Hcy is linked to disrupted methylation patterns [64], particularly related to genes regulating glucose metabolism, insulin secretion, and sensitivity, such as *MTNR1B, TCF7L2, CDKAL1, CDKN2A–CDKN2B,* and *HKDC1* [65], contributing to both maternal and adverse fetal outcomes. Paradoxically, while folate can lower homocysteine [66,67], this effect plateaus at high doses.

Regarding the previous evidence, FA excess increases Hcy because of MTHFR downregulation by DHFR saturation, which impairs Hcy remethylation. Notably, this remethylation process happens in a B12-dependent reaction by MTR [68]. Therefore, when there is a B12 deficiency, Hcy tends to be higher. MTR enzyme also uses 5-methyl-THF as a methyl donor in the reaction, so in the context of low B12, folate remains “trapped” in this methylated form; its accumulation in the nucleus has been linked to genomic instability, potentiating detrimental effects of FA high intake [69]. Recent literature shows that MTHFR C677T and A1298C contribute to GDM pathogenesis. The MTHFR 677 TT and 1298 AA genotypes are more prone to GDM dependent on one-carbon metabolism nutrients [70].

This might lead to functional folate deficiency despite high intake, homocysteine accumulation, and disrupted methylation patterns in GDM.

### 4.2. Methodological Considerations

This review followed PRISMA 2020 guidelines. We included only human studies indexed in major scientific databases. Study quality assessment was performed using the Newcastle–Ottawa Scale, with all studies rated as moderate to low bias risk, which enhances reliability in findings.

However, substantial heterogeneity among the included data, folic acid dose, timing, and GDM definitions limited comparability, so no metanalysis was performed. Almost all study designs were observational, which could result in potential residual confounding. Few cohorts adjusted for variables such as vitamin B12 status, dietary folate, or genetic variants affecting one-carbon metabolism, such as those found in *MTHFR* and *MTRR*. It is important to note that folic acid metabolism and response may vary depending on individual factors such as maternal body weight, nutritional status, age, ethnicity, and comorbidities. Most studies did not stratify results by these variables, which limits the applicability of the findings to broader populations. Most included studies were conducted in Chinese populations. Although these were derived from different provinces and hospitals, the concentration of data from a single country may limit generalizability to other ethnic and geographic populations.

## 5. Conclusions

This systematic review highlights a complex relationship between FA supplementation and the risk of GDM; while the standard FA dose (400 μg/day) remains essential for preventing neural tube defects, some studies suggest that no supplementation, high doses, or prolonged use—especially when started preconceptionally and in the presence of vitamin B12 deficiency—may increase GDM risk. Study heterogeneity limits definitive conclusions, but mechanistic evidence points to potential metabolic disturbances such as homocysteine accumulation, oxidative stress, and impaired insulin signaling. Given that FA fortification and supplementation are widespread practices globally, these findings suggest the need for individualized prenatal nutrition approaches. Therefore, we advocate that future research incorporates genetic, metabolic, and nutritional factors to better define optimal FA dosing and timing, aiming to balance fetal development benefits with minimized maternal metabolic risk.

## Figures and Tables

**Table 1:** Studies evaluating the association between gestational diabetes mellitus and folic acid supplementation.

Study (Year)	Design/GDM Diagnosis	Population/ Ethnicity	Folic Acid Exposure	GDM Outcome	Risk of Bias (NOS)
Li et al. (2015) [35]	Case-control with non-randomized controlled trial Not specified	2928 individualized c FA supp. 4884 controls no FA intervention. Chinese	Case: FA dosed 0.4–0.8 mg in different pregnancy periods according to MTHFR/MTRR risk level Control: No FA supplementation	GDM incidence was reduced in FA-supplemented group (0.27%) vs. control (3.24%, *p* < 0.05).	Low
Zhu et al. (2016) [27]	Prospective Cohort 75-g OGTT at 28 weeks	249 GDM cases 1689 normal pregnancies Chinese	Early pregnancy FA supplement use vs. none (timing not fully detailed)	GDM incidence: 12. 85% GDM risk: Increased with FA use in early pregnancy (first trimester, OR 2.25, 95% CI: 1.35–3.76)	Moderate
Lai et al. (2018) [28]	Prospective multi-ethnic cohort (GUSTO) 75-g OGTT at 26 weeks	913 pregnant women Chinese, Malay, Indian	Folate and B12 levels at 26 weeks used as proxy for high folate; supplementation nearly universal at standard dose.	GDM incidence: 18% GDM risk: Increased with high folate + low B12 status. OR 1.97 (95% CI 1.05–3.68).	Low
Huang et al. (2019) [29]	Prospective cohort study 75-g OGTT at 28 weeks	326 pregnant Chinese	FA supplementation ranged from 400 to 1200 μg/dose: 93.5% took 400 μg/d; 85% used FA alone, 15% used multivitamins. Duration: 7.7% for 6 mo, 2.1% >6 mo, 9.8% none.	GDM incidence: 10.1%. GDM risk: U-shaped association with FA duration. No FA or >90 d linked to higher GDM risk (OR = 3.45, 95% CI: 1.01–11.8); 1–60 d around conception had lowest risk.	Low
Cheng et al. (2019) [30]	Prospective cohort study 75-g OGTT at 24–28 weeks	950 mother- offspring pairs. Chinese	FA supp. 98.6%; pre-pregancy. 65.3% (≥3 mo: 30.8%), during pregnancy. 97.9% (≥3 mo: 73.9%)	GDM incidence: 10.2% GDM risk: increased with FA supp. ≥3 mo before pregnancy assoc. ARR 1.72 (95% CI: 1.17–2.53); ≥3 mo. supp. During pregnancy not assoc. after adj.: ARR 0.92 (95% CI: 0.52–1.65).	Low
Study (year)	Design/GDM diagnosis	Population/ ethnicity	Folic acid exposure	GDM outcome	Risk of Bias
Li et al. (2019) [31]	Prospective cohort study 75-g OGTT at 24–28 weeks	4353 pregnant women Chinese	Nonusers: no FA or <400 µg/d or <4 wk. FA400-S: 400–800 µg/d, <4 wk. preconceptionally and/or <16 wk. pregnancy. FA400-L: 400–800 µg/d, ≥4 wk. preconceptionally + ≥16 wk. during pregnancy. FA800-S: 800 µg/d, <4 wk. preconceptionally and/or <16 wk. pregnancy. FA800-L: 800 µg/d, ≥4 wk. preconceptionally+ ≥16 wk. pregnancy	GDM Incidence: 8.6% GDM risk: increased with High-dose FA (≥800 µg/d, ≥3 mo) assoc. (adj. OR =1.7, 95% CI: 1.30–3.36) vs. ≤400 µg/d or shorter use.	Moderate
Guo et al. (2022) [20]	Prospective cohort study Patient medical records	7552 pregnant women Chinese	MV+FA (mean dose 1.17 mg/d) FA alone (mean dose 1.10 mg/d) vs. no FA.	GDM incidence: 1.11% GDM risk: No significant association found. Not in early FA supp. nor elevated homocysteine had a significant link with GDM MTHFR 677 TT genotype did not influence GDM risk	Moderate
Chen et al. (2022) [32]	Prospective cohort study 75-g OGTT at 24–28 weeks	24,429 enrolled (2018–2021); only 1305 completed FA intake data and met inclusion criteria. Chinese	1305 women classified by FA dose (<400, 400–800, >800 µg/d) and duration (<3, >3 mo).	GMD incidence: 26.18% GDM risk: FA supp. linked to decreased risk: total OR 0.82 (95% CI: 0.70–0.95); 1-h PBG OR 0.80 (CI: 0.67–0.95); 2-h PBG OR 0.71 (CI: 0.60–0.85).	Moderate
Pazzagli et al. (2022) [19]	Retrospective cohort study Patient medical records	Two cohorts: Norway (2005–2018, n = 791,709) and Sweden (2006–2016, n = 1,112,817). Nordic cohorts	Self-reported FA: 68.0% (Norway), 22.9% (Sweden); prescribed FA (1–5 mg): 1.0% (Norway), 1.8% (Sweden); assumed self-reported FA ≈ 0.4 mg/d.	GDM incidence: NOR 3.2%. SWE 1.2% GMD risk: mixed. Self-reported FA: minimal effect (NOR OR 1.10, 95% CI: 1.06–1.14. SWE OR 0.89, 95% CI: 0.85–0.93); prescribed high-dose FA (5 mg) Increased GDM risk (NOR OR 1.33, 95% CI: 1.15–1.53. SWE OR 1.56, 95% CI: 1.41–1.74).	Low
Zhu et al. (2023) [36]	Matched case control 75-g OGTT at 24–28 weeks	Case–control (n = 162): 81 GDM vs. 81 non-GDM, age-matched (≤3 yr), parity-matched; serum folate measured at 24–28 gw. Chinese	FA intake (self-reported, n = 151): <400 µg (n = 96), 400–800 µg (n = 42), >800 µg (n = 13); duration: <24 wk (n = 112), >24 wk (n = 39); non-users (n = 11).	GDM risk increased in no FA users (aOR 7.25, 95% CI: 1.34–39.36) and those who used > 800 μg/day (aOR 4.20, 95% CI: 1.03–17.22) vs. <400 µg/d; 400–800 µg/d not assoc. (aOR =1.26, 95% CI: 0.56–2.84); only no FA increased risk vs. ≤24 wk (aOR 6.70, 95% CI: 1.22–36.77); no assoc. for dietary or serum folate.	Low
Li et al. (2023) [33]	Prospective cohort study 75-g OGTT at 24–28 weeks	Pregnant women at 4–14 weeks gestation (n = 2095); 372 with GDM, 1723 non GDM. Chinese	FA supplementation: <400 µg/d (GDM = 35, non-GDM = 186); ≥400 µg/d (GDM = 337, non-GDM = 1909). Pre-pregnancy BMI in FA-deficient supplements: <25 (n = 147), 25–<30 (n = 34), ≥30 (n = 5); in adequate FA-supplemented: <25 (n = 1573), 25–<30 (n = 279), ≥30 (n = 57).	GDM incidence: 17.76% GDM risk increased with BMI; highest in obese with FA < 400 µg/d (aOR 10.82, 95% CI: 1.69–69.45); FA ≥ 400 µg/d decreased risk but not BMI effect (aOR 3.57. 95% CI: 2.02–6.34); FA-deficient vs. FA-sufficient NS within BMI groups (ROR NS).	Moderate
Zheng et al. (2024) [34]	Prospective cohort study 75-g OGTT at 24–28 weeks	2032 pregnant women of whom 392 developed GDM. Chinese	FA intake pre-conception: <400 µg (n = 877), 400–799 µg (n = 671), ≥800 µg (n = 484); post-conception: <400 µg (n = 104), 400–799 µg (n = 1010), ≥800 µg (n = 918).	GDM incidence: 19.3% GDM risk: no association with FA intake or folate-related genotypes. Higher early pregnancy UMFA ≥P75 (aOR 1.36, 95% CI: 1.01–1.84) and ≥P90 (aOR 1.82, 95%, CI: 1.23–2.69), and homocysteine (Hcy) ≥P75 (aOR 1.40, 95% CI: 1.04–1.88) linked to GDM.	Low

Abbreviations: ARR, adjusted risk ratio; aOR, adjusted odds ratio; BMI, body mass index; B12, vitamin B12; CI, confidence interval; FA, folic acid; FA-D, FA-deficient supplementation; FA-S, FA-sufficient supplementation; GDM, gestational diabetes mellitus; Hcy, homocysteine; MV, multivitamin; NOS, Newcastle–Ottawa Scale; NS, not significant; OGTT, oral glucose tolerance test; OR, odds ratio; PBG, plasma blood glucose; ROR, relative odds ratio; UMFA, unmetabolized folic acid; mo., months; wk., week; d, day.

## Data Availability

No new data was created the data supporting this review is from published studies that have been properly cited. This review was registered in the PROSPERO database: https://www.crd.york.ac.uk/PROSPERO/view/CRD420251037606 accessed on 29 June 2025.

## Abbreviations

The following abbreviations are used in this manuscript:

FA	Folic acid
GDM	Gestational diabetes mellitus
B12	Vitamin B12
UMFA	Unmetabolized folic acid
DHFR	Dihydrofolate reductase
THF	Tetrahydrofolate
MTHFR	Methylenetetrahydrofolate reductase
MTR	Methionine synthase
MTRR	Methionine synthase reductase
SAM	S-adenosylmethionine
SAH	S-adenosylhomocysteine
Hcy	Homocysteine
CBS	Cystathionine β-synthase
CSE	Cystathionine γ-lyase
MAT	Methionine adenosyltransferase
MT	Methyltransferase
TYMS	Thymidylate synthase
NADPH	Nicotinamide adenine dinucleotide phosphate
OGTT	Oral glucose tolerance test
aOR	Adjusted odds ratio
ARR	Adjusted risk ratio
OR	Odds ratio
CI	Confidence interval
BMI	Body mass index
NOS	Newcastle–Ottawa Scale
ROR	Relative odds ratio
PBG	Plasma blood glucose
MV	Multivitamin
wk.	Week
D	Day
5-methyl-THF	5-methyltetrahydrofolate
IR	Insulin receptor
C-Hcy	Cysteine-homocysteinylation
ROS	Reactive oxygen species
T2DM	Type 2 diabetes mellitus
SNP	Single nucleotide polymorphism
